# A new species of the genus *Pareas* (Squamata, Pareidae) from Yunnan, China

**DOI:** 10.3897/zookeys.1011.59029

**Published:** 2021-01-21

**Authors:** Shuo Liu, Dingqi Rao

**Affiliations:** 1 Kunming Natural History Museum of Zoology, Kunming Institute of Zoology, Chinese Academy of Sciences, 32 Jiaochang Donglu, Kunming, Yunnan 650223, China Kunming Institute of Zoology, Chinese Academy of Sciences Kunming China; 2 Kunming Institute of Zoology, Chinese Academy of Sciences, 32 Jiaochang Donglu, Kunming, Yunnan 650223, China Kunming Institute of Zoology, Chinese Academy of Sciences Kunming China

**Keywords:** Molecular, morphological, snail-eating snake, taxonomy

## Abstract

A new species of *Pareas* is described from Yunnan Province, China, based on morphological comparisons and molecular data. Genetically, the new species is most closely related to the recently-described *Pareas geminatus*, for which we present new topotypic findings. The genetic divergence (uncorrected p-distance) of the cytb gene between the new species and congeners ranged from 6.14% to 24.68%. Morphologically, the new species can be distinguished from *P. geminatus* and all other congeners. Our work brings the total number of species within the genus *Pareas* to 22.

## Introduction

The family Pareidae Romer encompasses two subfamilies and four genera (Deepak et al. 2018; Uetz et al. 2020) and was once considered as a subfamily (called Pareatinae) of Colubridae (Smith 1943; Zhao et al. 1998; Zhao 2006). The taxonomy of the genus *Pareas* Wagler remains in a state of flux and species complexes with wide distributions and several lineages with an unclear taxonomic status remain especially challenging (Guo et al. 2011; Vogel 2015; You et al. 2015; Vogel et al. 2020; Wang et al. 2020). You et al. (2015) re-evaluated the taxonomic status of the *P. hamptoni-formosensis* complex from Taiwan, China, Ryukyus and adjacent regions, but the taxonomic status of the complex from mainland Chian was not involved. Wang et al. (2020) described *P. menglaensis* Wang, Che, Liu, Li, Jin, Jiang, Shi and Guo in the *P. carinatus-nuchalis* complex from Yunnan, China, but without any comments on the distribution of *P. carinatus* (Wagler) and described *P. mengziensis* Wang, Che, Liu, Li, Jin, Jiang, Shi and Guo without first resolving the historically taxonomic confusions of *P. yunnanensis* (Vogt) and *P. niger* (Pope). Vogel et al. (2020) re-assessed the taxonomy of the *P. margaritophorus*-*macularius* complex, re-validated two species and further re-confirmed the full species status of *P. macularius* Theobald in the complex. Vogel et al. (2020) underlined that the taxonomy of the genus *Pareas* has not yet been fully assessed, especially in widely-distributed taxa often representing complexes of cryptic or morphologically-similar species.

During our field research in Yunnan Province, China, from 2019 to 2020, some small and slender arboreal nocturnal snakes with blunt snouts and no mental groove and no teeth on the anterior part of maxillary, which could be assigned to the genus *Pareas*, were collected from Lancang County, Jiangcheng County and Kunming City. Morphological comparison and molecular analyses indicated that the specimens from Lancang County are distinct from all named species of the genus *Pareas* and we consequently described them as a new species.

## Materials and methods

Specimens were collected in the field. Photographs were taken to document the colour pattern in life prior to euthanasia. Liver tissues were stored in 99% ethanol and snakes were preserved in 75% ethanol. Specimens were deposited at Kunming Natural History Museum of Zoology, Kunming Institute of Zoology, Chinese Academy of Sciences (**KIZ**).

### Morphometrics

Measurements were taken to the nearest 1 mm with digital calipers. Paired meristic characters are given as left/right. The methodology of measurements and meristic counts followed Wang et al. (2020). The abbreviations of measurements and meristic counts are given below:

**DS** dorsal scale rows (counted at one head length behind head, at mid-body and at one head length before vent, respectively);

**InfL** infralabials;

**LoBO** loreal bordering orbit;

**Max** maxillary teeth;

**NED** number of enlarged dorsal scale rows at mid-body;

**NKD** number of keeled dorsal scale rows at anterior/middle/posterior of body;

**PosO** postoculars;

**PreO** preoculars;

**PrFBO** prefrontal bordering orbit;

**Sc** subcaudals;

**SPOF** subocular-postocular fused or not;

**SubO** suboculars;

**SupL** supralabials;

**SVL** snout-vent length (from tip of snout to posterior margin of cloacal plate);

**Tem** temporals;

**TL** tail length (from posterior margin of cloacal plate to tip of tail);

**Vs** ventrals.

For comparison, data for other species were taken from related publications (Boulenger 1900, 1905; Pope 1935; Zhao et al. 1998; Grossmann and Tillack 2003; Guo and Deng 2009; Guo et al. 2011; Loredo et al. 2013; Vogel 2015; You et al. 2015; Hauser 2017; Bhosale et al. 2020; Ding et al. 2020; Vogel et al. 2020; Wang et al. 2020). In addition, we examined the topotypic specimens of *Pareas niger* preserved in KIZ.

### Phylogenetic analyses

Molecular data were generated for two specimens from Lancang County, two specimens from Jiangcheng County and one specimen from Kunming City. Homologous sequences were obtained from GenBank. All new sequences have been deposited in GenBank. *Aplopeltura boa* (Boie), *Asthenodipsas laevis* (Boie) and *Xylophis captaini* Gower and Winkler were selected as outgroups, based on Wang et al. (2020). All the GenBank accession numbers for taxa used in this study are listed in Table 1.

**Table 1.** Sequences (cytb) used in phylogenetic analysis of this study.

Species	Locality	Voucher no.	GenBank no.
*Pareas andersonii*	Longchuan, Yunnan, China	CHS 015	MK201238
*Pareas atayal*	N. Cross-Is. Highway, Taiwan, China	HC 000618	JF827685
*Pareas boulengeri*	Jiangkou, Guizhou, China	GP 2923	MK135090
*Pareas carinatus*	Malaysia	KIZ 011972	MK135111
*Pareas chinensis*	Junlian, Sichuan, China	GP 2196	MK135088
*Pareas formosensis*	N. Cross-Is. Highway, Taiwan, China	NMNS 05632	KJ642130
*Pareas formosensis*	Hainan, China	YBU 12015	MK135068
*Pareas formosensis*	Leishan, Guizhou, China	YBU 12090	MK135074
*Pareas formosensis*	Guangxi, China	YBU 14508	MK135076
*Pareas formosensis*	Jingning, Zhejiang, China	GP 4581	MK135072
*Pareas geminatus*	Jiangcheng, Yunnan, China	CIB 118021	MW287068
*Pareas geminatus*	Jiangcheng, Yunnan, China	KIZ L2020020	MW436707
*Pareas geminatus*	Jiangcheng, Yunnan, China	KIZ L2020024	MW436708
*Pareas hamptoni*	Myanmar	YPX 18219	MK135077
*Pareas hamptoni*	Myanmar	YPX 18604	MK135078
*Pareas iwasakii*	Ishigaki Is., S. Ryukyu, Japan	I03-ISG1	KJ642158
*Pareas kaduri*	Lohit, Arunachal, India	BNHS 3574	MT188734
*Pareas komaii*	Lijia, Taidong, Taiwan, China	HC 000669	JF827687
*Pareas macularius*	Hainan, China	GP815	MK135101
*Pareas margaritophorus*	Cangwu, Guangxi, China	YBU 16061	MK135097
*Pareas menglaensis*	Mengla, Yunnan, China	YBU 14124	MK135114
*Pareas mengziensis*	Mengzi, Yunnan, China	GP 1294	MK135079
*Pareas mengziensis*	Mengzi, Yunnan, China	YBU 14251	MK135080
*Pareas mengziensis*	Mengzi, Yunnan, China	YBU 14252	MK135081
*Pareas mengziensis*	Mengzi, Yunnan, China	YBU 14253	MK135082
*Pareas mengziensis*	Mengzi, Yunnan, China	YBU 14288	MK135083
*Pareas mengziensis*	Kaiyuan, Yunnan, China	YBU 15100	MK135084
*Pareas mengziensis*	Kaiyuan, Yunnan, China	YBU 15114	MK135085
*Pareas modestus*	Tanhril, Aizawl, Mizoram, India	MZMU 1293	MT968773
*Pareas monticola*	Motuo, Xizang, China	KIZ 014167	MK135109
*Pareas niger*	Kunming, Yunnan, China	KIZ 059339	MW436706
*Pareas nigriceps*	Gaoligongshan, Yunnan, China	CHS 656	MK201455
*Pareas stanleyi*	Guangxi, China	GP 229	MK135086
*Pareas vindumi*	Lukpwi, Chipwi, Kachin, Myanmar	CAS 248147	MT968776
*Pareas xuelinensis* sp. nov.	Lancang, Yunnan, China	KIZ XL1	MW436709
*Pareas xuelinensis* sp. nov.	Lancang, Yunnan, China	KIZ XL2	MW436710
*Aplopeltura boa*	Malaysia	LSUHC 7248	KC916746
*Asthenodipsas laevis*	Peninsular Malaysia	LSUHC 10346	KC916749
*Xylophis captaini*	Kottayam, Kerala, India	BNHS 3376	MK340914

Total genomic DNA was extracted from liver tissues using the OMEGA DNA Kit (Omega Bio-Tek, Inc., Norcross, GA, USA). The sequences of the mitochondrial gene fragment, cytochrome b (cytb), were amplified by polymerase chain reaction (PCR) using primers L14910/H16064 (Burbrink et al. 2000). The double-stranded products were purified and sequenced at Genewiz Co. (Suzhou, China). Sequences were edited and manually managed using SeqMan in Lasergene 7.1 (DNASTAR Inc., Madison, WI, USA) and MEGA 7 (Kumar et al. 2016).

Sequences were aligned using ClustalW (Thompson et al. 1994) integrated in MEGA 7 with default parameters (Kumar et al. 2016). The genetic divergence (uncorrected p-distance) between species was calculated in MEGA 7 with the parameters Transitions + Transversions, Uniform rates and Pairwise deletion (Kumar et al. 2016). The substitution model GTR+I was selected in MODELTEST 3.7 (Posada and Crandall 1998). Bayesian Inference was performed in MrBayes 3.2.6 (Ronquist et al. 2012), based on the selected substitution model. Two runs were performed simultaneously with four Markov chains starting from a random tree. The chains were run for 1,000,000 generations and sampled every 100 generations. The first 25% of the sampled trees was discarded as burn-in after the standard deviation of split frequencies of the two runs was less than a value of 0.01 and then the remaining trees were used to create a 50% majority-rule consensus tree and to estimate Bayesian posterior probabilities. Maximum Likelihood analysis was performed in RaxmlGUI 2.0 (Silvestro and Michalak 2012) and nodal support was estimated by 1,000 rapid bootstrap replicates.

## Results

Meristic and mensural characters were noted for all examined specimens (Tables 2, 3). The three specimens collected from Lancang County could be distinguished from all other congeners. Morphological characters of the two specimens from Jiangcheng County agreed with *Pareas geminatus* Ding, Chen, Suwannapoom, Nguyen, Poyarkov and Vogel and morphological characters of the specimen collected from Kunming City agreed with the topotypic specimens of *P. niger*.

**Table 2.** Measurements (in mm) and scalation data of *Pareas xuelinensis* sp. nov. and *P. geminatus*. For abbreviations, see Materials and methods section. Paired meristic characters are given as left/right.

	*Pareas xuelinensis* sp. nov.			*Pareas geminatus*	
	KIZ XL1	KIZ XL2	KIZ XL3	KIZ L2020020	KIZ L2020024
	Holotype	Paratype	Paratype	Topotype	Topotype
SEX	♂	♂	♀	♂	♂
SVL	403	431	287	344	316
TL	132	145	94	96	87
PrFBO	Yes	Yes	Yes	Yes	Yes
PreO	1	1	1	1	1
PosO	Fused	Fused	Fused	Fused	Fused
SubO	Fused	Fused	Fused	Fused	Fused
SPOF	Yes	Yes	Yes	Yes	Yes
Tem	2+2+2/2+2+2	2+3+2/2+2+3	2+2+2/2+2+2	1+2+1/1+2+1	1+1+1/1+2+1
SupL	7/7	7/7	7/7	7/7	7/7
InfL	7/7	7/7	8/8	8/8	7/7
LoBO	No	No	No	No	No
Vs	188	182	183	184	183
Sc	89	87	93	73	74
Ds	15-15-15	15-15-15	15-15-15	15-15-15	15-15-15
NED	0	0	0	1	1
NKD	0-3-5	0-3-5	0-3-5	0-5-5	0-5-5
Max	7/6	6/7	8/8	6/5	5/5

**Table 3.** Measurements (in mm) and scalation data of *Pareas niger*. For abbreviations, see Materials and methods section. Paired meristic characters are given left/right.

	KIZ 059339	KIZ 76003	KIZ 790009	KIZ 82001	KIZ 90I0004
	Topotype	Topotype	Topotype	Topotype	Topotype
SEX	♂	♂	♂	♂	♂
SVL	192	364	396	384	383
TL	53	103	incomplete	99	92
PrFBO	Yes	Yes	Yes	Yes	Yes
PreO	1	1	1	1	1
PosO	Fused	Fused	Fused	Fused	Fused
SubO	Fused	Fused	Fused	Fused	Fused
SPOF	Yes	Yes	Yes	Yes	Yes
Tem	2+3+3/2+3+3	2+2+2/2+1+3	2+3+2/1+3+3	1+3+3/1+3+1	2+3+2/2+3+3
SupL	7/7	7/7	7/8	7/7	7/7
InfL	9/9	9/8	7/7	7/7	8/9
LoBO	No	No	No	No	No
Vs	154	169	172	163	161
Sc	55	66	incomplete	59	65
Ds	15-15-15	15-15-15	15-15-15	15-15-15	15-15-15
NED	0	0	0	1	3
NKD	0-0-0	0-5-5	0-5-5	0-0-3	0-5-5
Max	6/6	8/7	6/6	6/6	6/7

**Figure 1.**
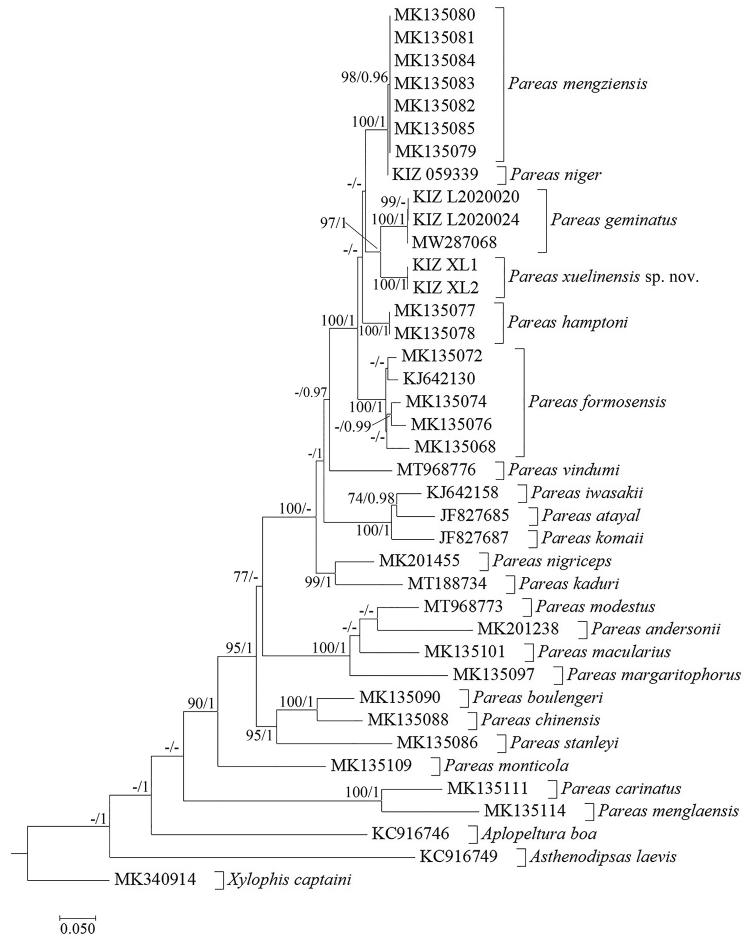
Maximum Likelihood phylogram of investigated members of *Pareas* and outgroups inferred from cytb gene. Numbers before slashes indicate bootstrap support for Maximum Likelihood analyses (only values above 70 are shown) and numbers after slashes indicate Bayesian posterior probabilities (only values above 0.9 are shown).

Maximum Likelihood analyses and Bayesian Inference showed similar results, the specimen form Kunming City clustered with *Pareas mengziensis*, the specimens from Jiangcheng County clustered with *P. geminatus* and the specimens from Lancang County formed a distinct lineage which is sister to *P. geminatus* with strong support (Fig. 1). The genetic divergence (uncorrected p-distance) between the lineage from Lancang County and *P. geminatus* was 6.14% (Table 4).

**Table 4.** Uncorrected p-distances (%) amongst the members of Pareidae, calculated from cytb gene sequences.

		1	2	3	4	5	6	7	8	9	10	11	12	13	14	15	16	17	18	19	20	21	22	23	24
1	*Pareas andersonii*																								
2	*Pareas atayal*	20.68																							
3	*Pareas boulengeri*	17.61	18.51																						
4	*Pareas carinatus*	23.93	23.20	22.56																					
5	*Pareas chinensis*	17.61	18.42	9.04	23.01																				
6	*Pareas formosensis*	18.84	15.10	16.60	24.02	16.88																			
7	*Pareas geminatus*	20.91	14.62	17.45	23.57	18.61	9.11																		
8	*Pareas hamptoni*	20.00	14.27	17.17	23.74	18.08	7.53	7.42																	
9	*Pareas iwasakii*	19.83	7.18	16.84	23.73	17.22	13.86	14.59	13.49																
10	*Pareas kaduri*	20.85	16.26	20.17	22.49	19.01	13.96	14.22	13.41	15.63															
11	*Pareas komaii*	18.97	8.66	18.14	23.94	18.23	14.40	15.09	14.46	7.94	16.58														
12	*Pareas macularius*	16.07	19.24	18.17	23.56	18.08	19.34	20.56	19.27	19.81	20.38	18.69													
13	*Pareas margaritophorus*	17.44	19.24	19.18	24.02	18.72	20.35	22.19	20.46	18.76	21.01	19.52	14.16												
14	*Pareas menglaensis*	22.91	23.39	23.56	14.06	25.02	24.24	23.04	23.56	23.44	24.82	23.94	24.29	24.93											
15	*Pareas mengziensis*	19.32	14.47	17.64	23.30	17.27	7.85	7.02	5.86	13.79	13.21	14.75	19.10	20.20	23.57										
16	*Pareas modestus*	12.82	18.42	19.18	24.11	19.09	19.98	20.34	19.63	19.33	19.54	17.77	10.87	13.88	24.02	19.01									
17	*Pareas monticola*	19.66	17.50	18.72	23.11	18.63	18.85	19.90	19.00	17.80	19.22	17.86	17.53	19.73	22.83	18.73	18.17								
18	*Pareas niger*	19.32	14.26	17.50	23.15	17.13	7.67	7.01	5.56	13.72	13.09	14.91	18.98	20.09	23.33	0.29	18.89	18.52							
19	*Pareas nigriceps*	17.61	16.07	16.92	22.91	16.07	12.92	13.39	12.65	16.07	10.43	16.24	20.00	17.95	23.08	12.65	16.41	19.15	12.48						
20	*Pareas stanleyi*	18.46	19.24	15.80	24.84	15.80	19.27	19.80	18.72	18.18	20.80	17.40	19.18	19.54	25.66	19.74	19.54	19.18	19.54	18.97					
21	*Pareas vindumi*	22.39	15.01	18.45	24.20	17.53	12.14	12.45	11.42	14.74	13.52	15.19	18.54	20.46	24.57	11.06	19.93	18.26	10.83	12.31	19.45				
22	*Pareas xuelinensis* sp. nov.	19.66	14.03	16.90	24.31	18.19	8.19	6.14	8.10	13.72	14.15	14.86	19.68	21.30	24.68	7.36	20.19	19.81	7.27	12.48	19.49	12.64			
23	*Aplopeltura boa*	23.25	23.16	21.43	23.78	21.43	23.37	24.56	23.78	23.27	24.39	23.67	23.98	24.49	23.16	23.38	22.45	21.12	23.37	23.25	22.65	23.78	25.00		
24	*Asthenodipsas laevis*	25.81	26.56	26.00	27.40	25.54	26.26	27.56	26.75	26.74	26.72	25.82	26.37	26.37	27.03	25.92	26.19	23.49	25.98	27.35	24.60	26.37	27.01	25.31	
25	*Xylophis captaini*	22.39	21.66	19.68	22.72	20.87	22.91	23.87	24.17	21.32	24.29	21.66	22.32	21.93	21.80	23.51	20.87	20.08	23.47	23.08	22.85	22.72	23.34	20.34	22.72

### Systematics

#### Pareas xuelinensis sp. nov.

Taxon classificationAnimalia

31B8BB56-7D91-5305-A75C-E3CC20342F93

http://zoobank.org/98E4DB90-251B-4C93-9B38-3A4C09001AD9

Figs 2 , 3 , 6A

##### Type material.

***Holotype*.**KIZ XL1, adult male, Xuelin Township, Lancang County, Pu’er City, Yunnan Province, China, 23°2'38"N, 99°32'35"E; 1840 m elevation, collected on 13 July 2019 by Shuo Liu.

***Paratypes*.**KIZ XL2, adult male and KIZ XL3, adult female, the same collection data as the holotype.

##### Diagnosis.

Single preocular; postocular fused with subocular; loreal not bordering orbit; prefrontal bordering orbit; fourth or fifth infralabial fused with second chin-shield; three chin-shield pairs; dorsal scales in 15 rows throughout; vertebral scales not enlarged; scales not keeled at the anterior part of the body, three rows of mid-dorsal scales keeled at the middle of the body, five rows of mid-dorsal scales keeled at the posterior of body; seven supralabials; seven or eight infralabials; cloaca undivided; ventral scales 182–188; subcaudals 87–93, paired.

**Figure 2.**
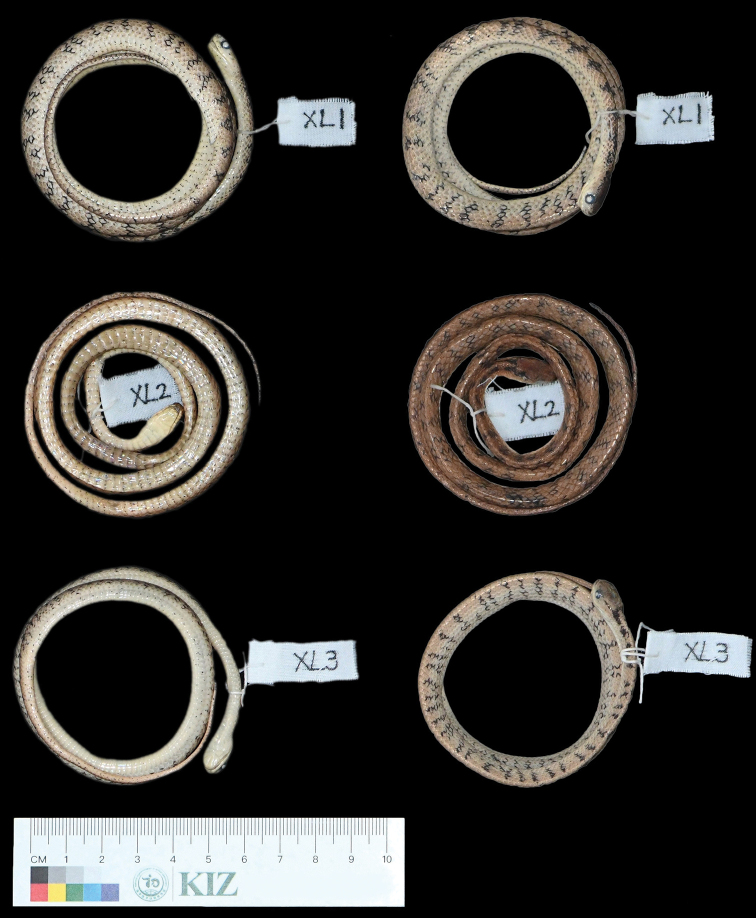
The type specimens of *Pareas xuelinensis* sp. nov. in preservative.

**Figure 3.**
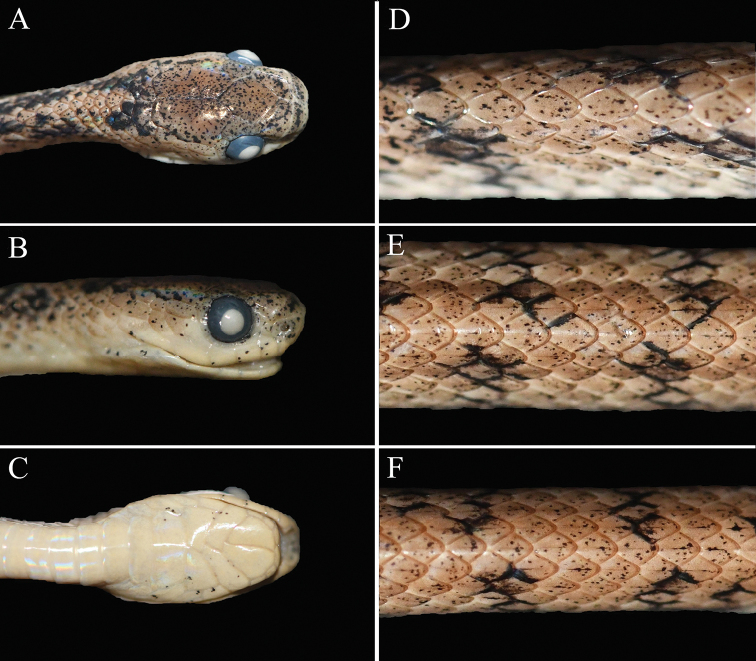
Holotype (KIZ XL1) of *Pareas xuelinensis* sp. nov. **A** dorsal view of the head **B** lateral view of the head **C** ventral view of the head **D** dorsal view of the anterior of body **E** dorsal view of the middle of body **F** dorsal view of the posterior of body.

##### Description of holotype.

Male, SVL 403 mm, TL 132 mm, TL/total length 0.25; body elongated; head distinct from neck; snout wide and blunt, projecting beyond lower jaw; body laterally compressed, vertebral ridge poorly developed. Rostral approximately as wide as high, almost invisible from above; nasals undivided; internasals elongated, much wider than long; prefrontals triangular, wider than long, bordering orbits; frontal shield-shaped, longer than wide; parietals large, longer than wide, median suture longer than frontal; single loreal, separated from eyes; single preocular; one relatively small supraocular, longer than wide; subocular and postocular fused into one thin elongated crescent-shaped scale; temporals 2+2+2 on both sides; seven supralabials on both sides, separating from eyes; seven infralabials on both sides, anterior-most in contact with its opposite between mental and anterior chin-shields, first four in contact with anterior chin-shields; fourth fused with second chin-shield; three chin-shields pairs, the first pair and the third pair triangle and almost equal size, the second pair elongate; ventral scales 188; cloaca undivided; subcaudals 89, paired; dorsal scales in 15 rows throughout, vertebral scales not enlarged, scales not keeled at anterior of body, three rows of mid-dorsal scales keeled at middle of body, five rows of mid-dorsal scales keeled at posterior of body; seven maxillary teeth on left side and six maxillary teeth on right side; hemi-penis in situ extending to the 19^th^ subcaudal.

##### Colouration in life.

Dorsal surface of head and body reddish-yellow with many black tiny spots on each scale; a thin black discontinuous postorbital stripe extending from postocular to neck, which is connected with its fellow on the opposite side by a thick black line which curves forward so as to almost touch the parietals; two thick black discontinuous stripes on neck followed the black curves forward line; many irregular longitudinal black stripes on the sides of body and tail, the stripes on different sides not connected to each other on the vertebrals; belly and ventral surface of head and tail yellow with sparse small black spots; iris reddish-yellow, pupil black.

##### Colouration in preservative.

The reddish-yellow dorsal surface of the head and body faded to yellowish-white; the yellow belly and ventral surface of head and tail faded to pale white; the iris changed to greyish-black from reddish-yellow and the pupil changed from black to white.

##### Variations.

Morphometric and meristic data for the type series are provided in Table 3. The paratype KIZ XL2 has 2+3+2 temporals on the left side and 2+2+3 temporals on the right side. The paratype KIZ XL3 has eight infralabials on both sides, first five being in contact with anterior chin-shields, fifth fused with second chin-shield.

**Figure 4.**
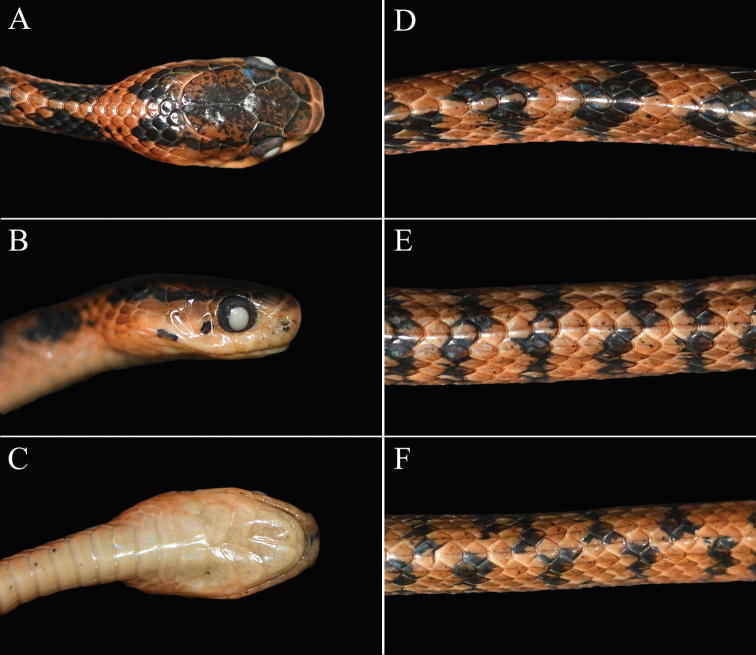
The specimen (KIZ L2020020) of *Pareas geminatus* collected from Jiangcheng County, Pu’er City, Yunnan Province, China **A** dorsal view of the head **B** lateral view of the head **C** ventral view of the head **D** dorsal view of the anterior of body **E** dorsal view of the middle of body **F** dorsal view of the posterior of body.

##### Etymology.

The specific epithet *xuelinensis* refers to Xuelin Township, the type locality of the new species.

##### Distribution.

This species is currently known only from the type locality Xuelin Township, Lancang County, Pu’er City, Yunnan Province, China. It is expected to be found in Myanmar.

##### Habitat.

Both the holotype and paratypes were found on the bushes beside a small road at night, surrounded by forest and farmland, with no river or stream nearby.

**Figure 5.**
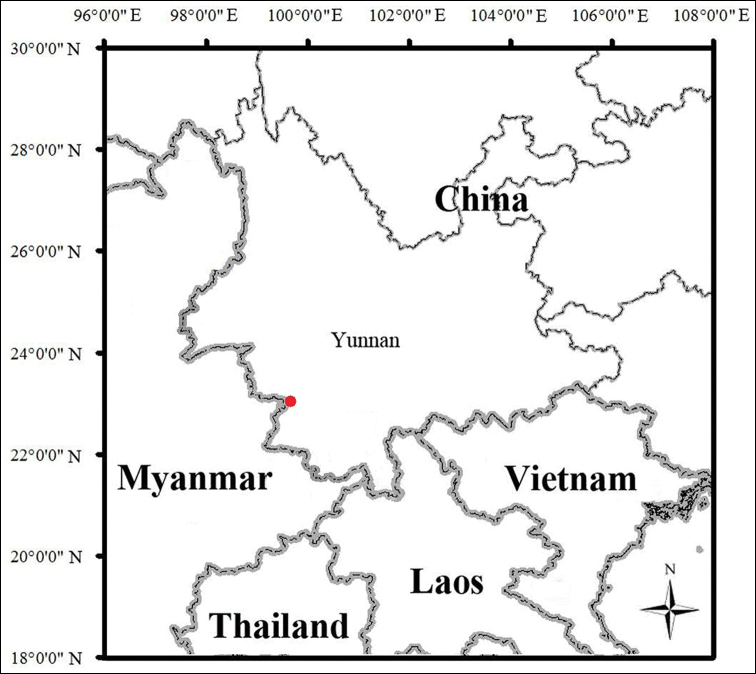
The type locality of *Pareas xuelinensis* sp. nov. (red dot) in Xuelin Township, Lancang County, Pu’er City, Yunnan Province, China.

##### Comparison.

*Pareas xuelinensis* sp. nov. can be distinguished from *P. andersonii* Boulenger, *P. atayal* You, Poyarkov & Lin *P. iwasakii* (Maki), *P. komaii* (Maki), *P. macularius* Theobald, *P. nigriceps* Guo & Deng and *P. stanleyi* (Boulenger) by 0–5 rows of mid-dorsal scales keeled (vs. 5–13 rows of mid-dorsal scales keeled); from *P. boulengeri* (Angel), *P. margaritophorus* (Jan), *P. monticola* (Cantor) and *P. vindumi* Vogel by three rows of mid-dorsal scales keeled at middle of body, five rows of mid-dorsal scales keeled at posterior of body (vs. all dorsal scales smooth); from *P. carinatus*, *P. menglaensis* and *P. nuchalis* (Boulenger) by subocular and postocular fused into one thin elongated crescent-shaped scale (vs. two or three distinct narrow suboculars); from *P. chinensis* (Barbour) and *P. modestus* Theobald by more ventral scales (182–188 vs. 136–176); and from *P. formosensis* (Van Denburgh) and *P. kaduri* Bhosale, Phansalkar, Sawant, Gowande, Patel and Mirza by vertebral scales not enlarged (vs. vertebral scales enlarged).

**Figure 6.**
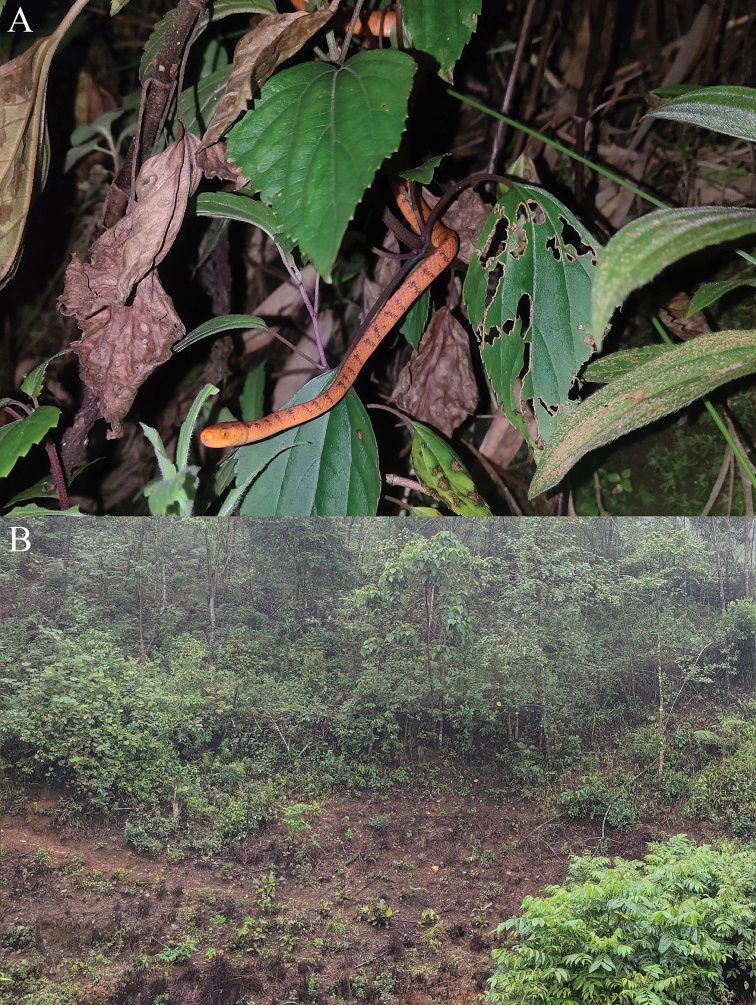
*Pareas xuelinensis* sp. nov. in life (**A**) and the habitat of *Pareas xuelinensis* sp. nov. at the type locality (**B**).

*Pareas xuelinensis* sp. nov. can be distinguished from *P. geminatus* by vertebral scales not enlarged (vs. vertebral scales enlarged), three rows of mid-dorsal scales keeled at middle of body (vs. five rows of mid-dorsal scales keeled at middle of body), fourth or fifth infralabial fused with the second chin-shield (vs. infralabials not fused with chin-shield), temporals 2+2+2 or 2+3+3 (vs. 1+2+1 or 1+1+1) and no black spot on each side of head (vs. having two black spots on each side of head).

*Pareas xuelinensis* sp. nov. can be distinguished from *P. hamptoni* (Boulenger) by vertebral scales not enlarged (vs. vertebral scales enlarged), temporals 2+2 or 2+3 (vs. 1+2) and less ventral scales (182–188 vs. 202).

*Pareas xuelinensis* sp. nov. can be distinguished from *P. mengziensis* by vertebral scales not enlarged (vs. vertebral scales enlarged), 0–5 rows of mid-dorsal scales keeled (vs. 3–9 rows of mid-dorsal scales keeled), having more ventral scales (182–188 vs. 167–173), more subcaudals (87–93 vs. 54–61) and the dorsal surface of head and body reddish-yellow (vs. the dorsal surface of head and body solid black).

*Pareas xuelinensis* sp. nov. can be distinguished from *P. niger* by having more ventral scales (182–188 vs. 154–172), more subcaudals (87–93 vs. 55–66) and the dorsal surface of head and body reddish-yellow (vs. the dorsal surface of head and body solid black).

*Pareas xuelinensis* sp. nov. can be distinguished from *P. yunnanensis* by the loreal separating from the eye (vs. the point of the large loreal touching the eye), vertebral scales not enlarged (vs. vertebral scales enlarged), and 0–5 rows of mid-dorsal scales keeled (vs. six rows of dorsal scales keeled).

**Figure 7.**
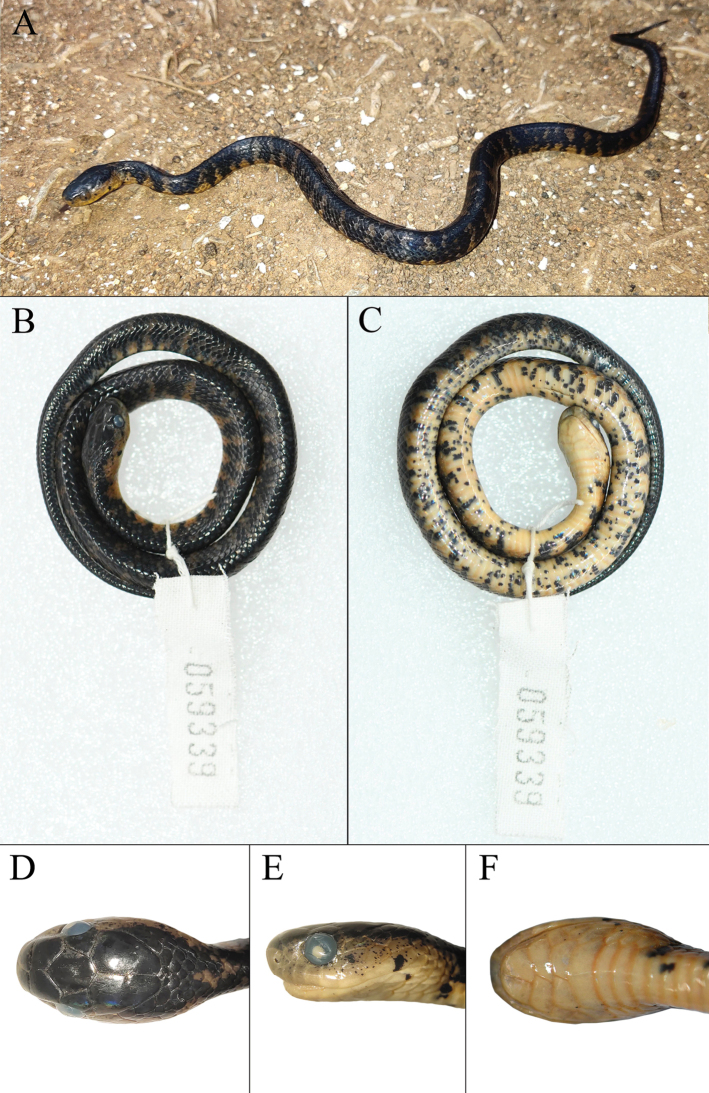
The specimen (KIZ 059339) of *Pareas niger* collected from Kunming, Yunnan, China **A** in life **B** dorsal view in preservative **C** ventral view in preservative **D** dorsal view of the head **E** lateral view of the head **F** ventral view of the head.

## Discussion

*Amblycephalus niger* (now *Pareas niger*) was described by Pope (1928) from Yunnanfu (now Kunming). *Pareas niger* was considered as synonyms of *P. chinensis*, *P. yunnanensis* and *P. komaii*, successively (Sichuan Institute of Biology 1977; Rao and Yang 1992; Uetz et al. 2020). We compared the morphometric and meristic data of *P. niger*, *P. chinensis*, *P. komaii* and *P. yunnanensis* (Table 5) and found that *P. niger* can be distinguished from *P. chinensis*, *P. komaii* and *P. yunnanensis*, so we consider *P. niger* to represent a valid species. During August 2019, we collected one specimen (voucher: KIZ 059339; Fig. 7) of *Pareas* with a solid black dorsal surface on the head and body and no enlarged mid-dorsal scales from Changchong Mountain in Kunming City, its morphological characters agreeing with the original description of *P. niger*, except that it has nine infralabials, which agreed with the original description of *P. mengziensis*. Molecularly, the specimen from Kunming was clustered together with *P. mengziensis* and the genetic divergence (uncorrected p-distance) between the specimen from Kunming and *P. mengziensis* was only 0.29%. After checking the topotypic specimens of *P. niger* preserved in KIZ, we found that the infralabials of *P. niger* range from seven to nine and one or three rows of mid-dorsal scales of several individuals are slightly enlarged. This means that the specimen (KIZ 059339) from Kunming should belong to *P. niger* and it is not appropriate to distinguish *P. mengziensis* and *P. niger* either morphologically or molecularly; therefore, we consider *P. mengziensis* and *P. niger* as the same species, *P. mengziensis* being a synonym of *P. niger*.

**Table 5.** Comparisons of morphometric and meristic data for *Pareas niger*, *P. chinensis*, *P. komaii* and *P. yunnanensis*. The data for *P. chinensis*, *P. komaii* and *P. yunnanensis* were obtained from the original descriptions and the subsequent descriptions of the type specimens (Barbour 1912; Vogt 1922; Maki 1931; Pope 1935). “?” = data not available.

	*Pareas niger*	*Pareas chinensis*	*Pareas komaii*	*Pareas yunnanensis*
SVL	192–396	?	430–470	385–410
TL	53–103	?	130	95–100
TL/Total length	0.19–0.22	?	0.20–0.25	0.20
PrFBO	Yes	Yes	Yes	Yes
PreO	1	2	1	2
PosO	Fused	1–2	1	1–2
SubO	Fused	0	1	1
SPOF	Yes	No	No	No
Anterior temporals	1–2	2	2	2
Posterior temporals	1–3	3	3	2–3
SupL	7–8	7	7	6–7
InfL	7–9	?	7	?
LoBO	No	No	No	Yes
Vs	154–172	180	175–179	171–176
Sc	55–66	60	72–75	64–65
Ds	15-15-15	15-15-15	15-15-15	15-15-15
NED	0–3	3	1	1
NKD	0–5	0	3–13	6
Max	6–8	5–6	?	?

## Supplementary Material

XML Treatment for Pareas xuelinensis
